# Neuropharmacological safety evaluation of jigrine: A polyherbal hepatoprotective formulation

**DOI:** 10.4103/0975-7406.72134

**Published:** 2010

**Authors:** A. K. Najmi, K. K. Pillai, S. N. Pal, M. Akhtar, M. Mujeeb, A. Aftab

**Affiliations:** Department of Pharmacology, India; 1 WHO, 20, Avenue Appia, Geneva 27, Switzerland; 2 Pharmacognosy, Faculty of Pharmacy, Jamia Hamdard, New Delhi 110062, India

**Keywords:** Hepatoprotective, jigrine, neuropharmacological, safety, unani

## Abstract

**Objective:**

Jigrine is a herbal hepatoprotective formulation containing aqueous extracts of 14 medicinal plants. Present study was designed to evaluate *per se* neuropharmacological effects of jigrine in mice.

**Materials and Methods:**

Jigrine was evaluated in a number of pharmacological test paradigms, viz. open field arena, actophotometer, hole board, rotarod, traction test, grip strength test, spontaneous alternation behavior, passive avoidance task, and phenobarbital sleeping time.

**Results and Conclusions:**

Jigrine pretreatment (1 and 2 ml/kg, p.o.) did not produce any significant effect as compared to normal saline treated animals and was found to be free from any acute undesirable central effects at these two dose levels.

Herbal drugs are widely used for the treatment of various diseases. Although herbal drugs often contain highly active pharmacological compounds but much importance is not given to their safety evaluation, may be due to a popular notion “anything herbal is safe.” Lately, with recent increasing interest in traditional or herbal drugs for the prevention and treatment of various disorders, there is also increasing concern about the safety of traditional, herbal product based medicines.[1]

Jigrine is a polypharmaceutical herbal formulation containing aqueous extracts of 14 medicinal plants used for liver ailments in traditional Indian system of medicine (Unani) [Table 1]. Few studies are reported for its formulation,[2] mechanism of hepatoprotective action,[3–6] and anti-inflammatory activity.[7] Present study was designed to evaluate *per se* neuropharmacological effects of jigrine in mice.

**Table 1:** Medicinal plant ingredients of Jigrine (a phytopharmaceutical formulation)

Botanical name	Common name part used	Unani name	Family
*Cichorium intybus* Linn	Chicory leave Kasni	Tukhme	Compositeae
*Tamarix dioica* Roxb	Tamarisk leave	Jhau	Tamaricaceae
*Solanum nigrum* Linn	Black nightshade fruit	Makoh	Solanaceae
*Rheum emodi* wall	Indian rhubarb Rhizo Chini me	Revand	Polygonaceae
*Rubia cordifolia* Linn	Indian madder root	Majeeth	Rubiaceae
*Vitex negundo* Linn	Nisinda whole shrub	Sambhalu	Verbenaceae
*Cassia occidentalis* Linn	Coffee senna	Kasaundi	Caesalpiniaceae leave
*Foeniculum vulgare* Mill	Fennel fruit	Sonf	Umbellifereae
*Cuscuta reflexa* Roxb	Amarvella	Tukhme	Convolvulaceae seed Kasoos
*Careya arborea* Roxb	Wild guava	Baokhamba	Barringtoniaceae fruit
*Phyllanthus niruri* Linn	Jaramla leave	Bhui amla	Euphorbiaceae
*Plantago major* Linn	Isphagol leave	Bartang	Plantaginaceae
*Rosa damascena* Linn	Damask rose flower	Gul-e-surkh	Rosaceae
*Solanum xanthocarpum*	Yellow berries schrad & wendl.	Katheli	Solanaceae

## Materials and Methods

### Animals

Albino mice weighing 20–30 g were used for the study. Animals were supplied by Central Animal House Facility of Jamia Hamdard (Hamdard University) and kept under standard laboratory conditions in 12 h light/dark cycle at 25±2°C. Animals were provided with pellet diet (Lipton, India) and water *ad libitum*. All the procedures carried out on animals were approved by institutional animal ethics committee (JHAEC).

### Experimental protocol

For each of the following tests, mice were divided into three groups of six animals each. Group I served as normal control and received normal saline (1 ml/kg, p.o.). Groups II and III received jigrine 1 and 2 ml/kg, p.o., respectively.

#### Actophotometer activity

Mice were observed for their spontaneous motor activity by placing them individually in the actophotometer and number of beam breaks was recorded for 6 min. This apparatus consisted of a square chamber and the activity of the animals was recorded by light beams passing through the chamber and connected to photoelectric cells. The activity was recorded at 0 time and 30, 60, 90, and 120 min after their respective treatments.[8] The actophotometer was cleaned with cotton swab dipped in 70% alcohol in between the observations.

#### Open field activity

Mice were observed for open field activity as per the method of Tricklebank *et al*.[9] for 6 min 1 h after their respective treatments. Mice were placed singly into the open field arena and the following behavioral parameters were scored: ambulation (number of areas entered with all four paws); frequency of rearing, and frequency of grooming. Fecal pellets were removed after every occupation and floor wiped with clean damp tissues after every occupation. The open field consisted of a circular arena 85 cm in diameter, divided into 25 segments of approximately equal area by black painted lines on the white floor. The arena was bound by a wall 30 cm high.

#### Hole board activity

Mice were observed on the wooden hole board at 0 time (before treatment) and 60 and 120 min after their respective treatments.

The wooden hole board apparatus consisted of 60 ×30 cm dimensions with 16 evenly placed holes and painted gray. The number of times the mice dipped their heads in holes during a 3-min trial was counted.[10]

#### Rotarod activity

Mice were trained to stay on the rotating rod of the rotarod (20 rpm) for 3 min. Trained mice were randomly divided into three groups as described above. All the animals were placed on the rotating rod (three animals at a time, one in each of the three compartments) 60 and 120 min after their respective treatments and the length of time they remained on the rotating rod was recorded.[11]

#### Traction test

This test was carried out as per the procedure reported by Anca *et al*.[11] Animals were suspended by their hind legs from a taut metal wire and the time taken to bring their front paws up to the wire was recorded. Animals were considered to have passed or failed the test according to whether this did or did not occur within 5 sec. Failure was considered to be synonymous with muscle relaxation.[11,12]

#### Grip strength test

Grip strength of animals in all the 3 groups was measured as an indicator of neuromuscular function after 1hour of their respective treatments. The grip strength meter was positioned horizontally. Mice were held by the tail and lowered toward the apparatus. Mice were allowed to grasp smooth metal wire mesh (fore limbs only) and were then pulled backward in the horizontal plane. The force applied to the bar at the moment the grasp was released was recorded as the peak tension (kg). The test was repeated five times within the same session and the highest value from the five trials was recorded as grip strength for that animal.[13]

#### Spontaneous alternation behavior

Spontaneous alternation behavior in mice was assessed by using a cross-maze. The maze was made up of plywood (painted gray) and consisted of symmetrical arms (23.5 cm long and 8 cm wide) with 10 cm high sidewalls. The arms extended from a central platform (8 × 8 cm) at a height of 50 cm above the floor and were labeled A, B, C, D. Mice were allowed to traverse the maze freely for 6 min after being placed on the central platform. The number and sequence of arm entries were recorded for calculation of a “percent alternation score.” An alternation was defined as entry into four consecutive arms on overlapping quintuple sets. Five consecutive arm choices within the set of arm choices made up quintuple sets. A quintuple set consisting of arm choices, ABDAC, was considered an alternation. A quintuple set consisting of arm choices, ABDAB, was not considered an alternation. Using this procedure, possible alternation sequences are equal to the number of arm entries minus 4. The percent alternation score is equal to the ratio of (actual alternation/possible alternation) × 100.[14]

#### Passive avoidance task

The method of Papazova *et al*.[15] was used with slight modifications. An inverted petridish was placed in the center of a continuous avoidance apparatus to serve as shock free zone (SFZ). Mice were placed in the SFZ and on stepping down the SFZ were given an electric shock of 20 mV through the grid floor. Animals were trained to remain on the SFZ for at least 60 sec. Mice that did not get trained in upto five trials were rejected. Observations were made for retention of learning (memory) for 10 min 24 h after the initial trials. One hour prior to retention observations, animals were given their respective treatment in three groups. The following retention parameters were recorded: step down latency (SDL) in seconds, step down errors (SDE) as the number of times the animals stepped down from SFZ, and the total time spent in shock zone (TSZ) in seconds.[16]

#### Pentobarbital sleeping time

One hour after their respective treatments, all the animals received pentobarbitobne (40 mg/kg, i.p.). Time interval between administration of pentobarbital until the loss of righting reflex was recorded as the onset of sleep while the time from the loss of righting reflex to recovery was recorded as the sleeping time.[17]

### Statistical analysis

Results are expressed as mean±SEM. Total variation present in a set of data was estimated by ANOVA followed by Dunnet’s post hoc test. *P*< 0.05 was considered significant.

## Results and Discussion

Jigrine was evaluated for its per se effects on locomotor activity, open field activity, hole board activity, rotarod performance, muscle relaxation, muscle grip strength, spontaneous alternation behavior, and sodium pentobarbital induced sleeping time in mice to assess its neuropharmacological safety profile. Locomotor[8] and open field[9] activities are done to evaluate the effect on motor activity of the animals. Hole board[10] activity is done to see the effect on anxiety of the animals. Rotarod,[11] muscle relaxation,[12] and grip strength[13] activities are done to check the muscular strength. Spontaneous alternation behavior[14] and passive avoidance[15] studies are done to find out the effect on learning and memory. Sodium pentobarbital[17] induced sleeping time study is done to check the central depressant effects of the substance evaluated. Jigrine pretreatment at the dose levels of 1 and 2 ml/kg, p.o. (groups II and III) in mice did not produce any significant change [Tables 2–10] in any of the above test paradigms as compared to normal saline treated animals (Group I). On the basis of these results, it may be suggested that jigrine has no undesirable central effects per se in mice for short-term treatment at these two doses.

**Table 2:** Per se effect of jigrine in two dose levels on locomotor activity of mice

Group	No. of beam breaks					
	0 min	30 min	60 min	90 min	120 min
I	566.16±52.78	330.5±45.822	232.33±61.01	291.16±44.73	270.83±48.05
II	528.5±40.48 ^ns^	379.0±37.04 ^ns^	274.0±38.28 ^ns^	239.0±41.05^ns^	262.33±28.97^ns^
III	611.66±66.59^ns^	454.056.04 ^ns^	458.0±57.25*	392.66±70.62 ^ns^	316.0±63.61^ns^
F ratio	0.582	1.761	5.110	2.100	0.346

Data expressed as mean ± SEM, n=6. Significance of difference was evaluated with respect to group I (control) at each time interval by one-way ANOVA followed by Dunnet’s post hoc test

(P* < 0.05, ns=nonsignificant)

**Table 3:** Per se effect of jigrine on open field activity in mice

Group	Ambulation	Grooming	Rearing
I	136.0±20.85	5.33±2.94	33.66±12.19
II	129.5±18.20^ns^	4.16±1.72^ns^	30.012.74^ns^
III	143.83±9.78^ns^	4.83±2.31^ns^	36.337.14^ns^
F ratio	0.174	0.362	0.502

Values indicate numbers of ambulation, grooming, and rearing. Data expressed as mean ± SEM, *n*=6. Significance of difference was evaluated with respect to group I by one-way ANOVA followed by Dunnet’s post hoc test. ns=nonsignificant

**Table 4:** Per se effect of jigrine on exploratory behavior of mice in hole-board test

Group	No. of nose pokes		
	0 min	60 min	120 min
I	19.5±1.31 ^n^	15.33±2.27	10.0±1.29
II	14.16±1.35 ^ns^	14.66±3.97 ^ns^	7.0±1.75 ^ns^
III	17.83±1.47^ns^	12.16±2.61 ^ns^	7.66±1.90 ^ns^
F ratio	1.576	0.3009	0.888

Data expressed as mean ± SEM, *n*=6. Significance of difference was evaluated with respect to group I (control) at each time interval by oneway ANOVA followed by Dunnet’s post hoc test. ns=nonsignificant

**Table 5:** Per se effect of jigrine on rotarod performance in mice

Group	Endurance time (sec)		
	Cut off time (sec)	60 min	120 min
I	180	180.0±0.0	178.16±1.83
II	180	178.0±1.33^ns^	177.66±1.96 ^ns^
III	180	176.83±3.16 ^ns^	180.0±0.0 ^ns^
F ratio		0.642	0.628

Data expressed as mean ± SEM, *n*=6. Significance of difference was evaluated with respect to group I (control) at each time interval by oneway ANOVA followed by Dunnet’s post hoc test. ns=nonsignificant

**Table 6:** Per se effect of jigrine on muscle relaxation (traction test)

Group	Positive number	
	60 min	120 min
I	6.0±0	6.0±0
II	6.0±0	6.0±0
III	6.0±0	6.0±0

Each value represents the mean±SEM number of animals passing the traction test. Data expressed as mean ± SEM, *n*=6

**Table 7:** Per se effect of jigrine on muscle grip strength in mice

Group	Grip strength (kg)
I	0.145±0.008
II	0.157±0.013^ns^
III	0.158±0.017^ns^
F ratio	1.674

Data expressed as mean ± SEM, *n*=6. Significance of difference was evaluated with respect to group I by one-way ANOVA followed by Dunnet’s post hoc test. ns=nonsignificant

**Table 8:** Per se effect of jigrine on spontaneous alternation behavior in mice

Group	% Alternation score	No. of arm entries
I	59.44±4.81	24.5±6.50
II	73.64±4.67ns	26.5±4.69^ns^
III	86.23±4.17*	28.66±6.77^ns^
F ratio	8.629	0.692

Data expressed as mean ± SEM, *n*=6. Significance of difference was evaluated with respect to group I by one-way ANOVA followed by Dunnet’s post hoc test. *P*<0.05, ns=nonsignificant

**Table 9:** Per se effect of jigrine on memory retention performance in passive avoidance task in mice

Group	SDL (sec)	SDE (number)	TSZ (sec)
I	14.33±3.31	2.0±0.85	17.33±4.08
II	18.16±4.09 ^ns^	1.66±0.76 ^ns^	19.5±7.42 ^ns^
III	15.00±2.79 ^ns^	1.83±0.40 ^ns^	14.667.27 ^ns^

Data expressed as mean ± SEM, *n*=6. Significance of difference was evaluated with respect to group I by one-way ANOVA followed by Dunnet’s post hoc test. ns=nonsignificant

**Table 10:** Per se effect of jigrine on sodium pentobarbital induced sleeping time

Group	Onset of sleep (min)	Duration of sleep (min)
I	2.43±0.17	56.91±4.24
II	2.99±0.42^ns^	50.67±1.70^ns^
III	2.74±0.12^ns^	53.94±2.54^ns^
F ratio	1.074	1.067

Data expressed as mean ± SEM, *n*=6. Significance of difference was evaluated with respect to group I (control) by one-way ANOVA followed by Dunnet’s post hoc test. ns=nonsignificant
